# Factors associated with the uptake of cataract surgery and interventions to improve uptake in low- and middle-income countries: A systematic review

**DOI:** 10.1371/journal.pone.0235699

**Published:** 2020-07-09

**Authors:** Eunice Wandia Mailu, Bhavisha Virendrakumar, Stevens Bechange, Emma Jolley, Elena Schmidt

**Affiliations:** 1 Sightsavers, Nairobi, Kenya; 2 Sightsavers, Chippenham, United Kingdom; 3 Sightsavers, Kampala, Uganda; Charles Sturt University, AUSTRALIA

## Abstract

Despite significant evidence around barriers hindering timely access to cataract surgery in low- and middle-income countries (LMICs), little is known about the strategies necessary to overcome them and the factors associated with improved access. Despite significant evidence that certain groups, women for example, experience disproportionate difficulties in access, little is known about how to improve the situation for them. Two reviews were conducted recently: Ramke et al., 2018 reported experimental and quasi-experimental evaluations of interventions to improve access of cataract surgical services, and Mercer et al., 2019 investigated interventions to improve gender equity. The aim of this systematic review was to collate, appraise and synthesise evidence from studies on factors associated with uptake of cataract surgery and strategies to improve the uptake in LMICs. We performed a literature search of five electronic databases, google scholar and a detailed reference review. The review identified several strategies that have been suggested to improve uptake of cataract surgery including surgical awareness campaigns; use of successfully operated persons as champions; removal of patient direct and indirect costs; regular community outreach; and ensuring high quality surgeries. Our findings provide the basis for the development of a targeted combination of interventions to improve access and ensure interventions which address barriers are included in planning cataract surgical services. Future research should seek to examine the effectiveness of these strategies and identify other relevant factors associated with intervention effects.

## Introduction

Cataract occurs when the normal clear lens of the eye becomes cloudy leading to gradual, progressive loss of vision often in both eyes [1]. Cataract is a major cause of visual impairment globally, affecting 20 million people who are blind [1, 2] and another 65 million who have moderate or severe visual impairment. Women, people from poorer households, and those living in low and middle-income countries (LMICs) are disproportionately affected by visual loss from cataract [3–5].

Cataracts are primarily an age related disorder, however other factors may increase the risk of the disease, including corticosteroid use, smoking, exposure to ultraviolet-light, and other morbidities, such as diabetes mellitus [6–8]. Although cataracts cannot be prevented, a cost-effective surgery can restore vision [7]. Studies have suggested that a successful cataract surgery has a positive impact on individuals including their quality of life and ability to return to economic activities [9]. Despite technical advances in the management of cataract, the volume of cataract surgeries in many LMICs remains low. This can be attributed to a combination of supply-side (provider) and demand-side (patient) factors. Supply-side factors may include poor infrastructure, inadequate human resources and inefficiencies within the system [10]. Demand-side factors may include insufficient community awareness, fear of surgery, cultural beliefs and inability to travel to healthcare facilities [11, 12].

In contemporary theories of demand and supply [13], the important starting proposition is that different people have different needs that are expressed as wants, which, in combination with purchasing power, constitute the demand. It is generally agreed that in healthcare, there are various reasons as to why healthcare providers cannot effectively respond to every single patient want, the task is therefore to segment demand into homogeneous categories following a certain logic [14]. Once the segmentation is complete, the supply side can be organized to deliver offerings to the chosen segments [15]. Identifying different factors driving the demand for eye care services is therefore critical for a better understanding of eye health production function and maximising the productivity of eye care services.

Despite the available evidence on barriers to cataract services [12, 16–18], there remains a need to better understand how these barriers affect populations in different contexts. There is also an urgent need to identify interventions that work to address these barriers and increase surgery uptake [19–22]. Several studies have been conducted to identify such interventions, and in 2018, Ramke et al published a systematic review, which examined strategies to reduce eye health inequalities through cataract interventions focusing on disadvantaged groups [23]. The eligibility criteria for their review included randomised controlled trials (RCTs), quasi-experimental studies, before and after designs and time-series. Out of 2,865 records identified in the search, only two studies, both conducted in China, met the inclusion criteria. Another recent systematic review by Mercer et al (2019) examined specific interventions to reduce gender inequity. Out of 3,250 unique records, 13 studies (4 RCTs and 9 observational) were included. This review however focused on a number of eye conditions and only five studies were specific to age-related cataract. One study was rated as moderate quality, the remaining studies were low quality [24].

In this systematic review, we aimed to synthesize all available literature on the uptake of age-related cataract surgery and the specific factors associated with uptake and interventions aiming to improve it.

The following research questions were addressed in this review:

1. What is the reported uptake of cataract surgery in LMICs?
2. Which factors are associated with the uptake of cataract surgery in LMICs?
3. Which interventions have been reported to improve the uptake of cataract surgery in LMICs?

## Materials and methods

The review is reported according to the Preferred Reporting Items for Systematic Reviews and Meta-Analyses (PRISMA) guidelines [25].

### Outcome measures

The primary outcome measure of interest was cataract surgical uptake (CSU). This is defined as the proportion of people who, having been referred to surgery, have undergone surgery. This is most often measured within cohorts of cataract patients identified through screening activities or in cohorts of patients diagnosed with cataract, some of whom would have had a surgery and others would have not. Review authors conducted literature searches specifically for studies related to this outcome measure. If studies reported factors associated with the primary outcome or interventions aiming to change it, then these were also extracted and synthesised. In this review, we excluded studies that reported cataract surgical coverage, that is patients or eyes that have been operated for cataract out of those with operable cataract. This outcome is usually measured in cross-sectional surveys, such as Rapid Assessments of Avoidable Blindness (RAABs). In such surveys however it is unclear whether patients are aware of their need or of the opportunities available on the supply side to address it. Thus, their demand for healthcare cannot be treated in the same way as patients diagnosed with cataract and informed about cataract surgery options. In addition, only papers published after January 2000 were included, as significant advances to surgical techniques occurred in the 1990s, which impacted on both the supply and demand for cataract surgeries [26].

### Search strategies

Two review authors (EM and BV) independently conducted a search of relevant bibliographic databases including MEDLINE, CENTRAL, EMBASE, LILACS, and ISRCTN. Search terms used a combination of key words such as ‘cataract surgery’ and ‘facilitators’ (S1 Appendix). The cataract evidence gap map [27] and google scholar were also searched for further studies not identified through the databases. In addition, references of the included studies were screened to identify grey literature and to ensure that all relevant studies were included in the review.

### Inclusion and exclusion criteria

Inclusion criteria were studies a) involving participants with age-related cataract b) containing primary data on uptake of surgery, c) conducted in LMICs based on the World Bank classification, d) written in English, Portuguese and French; and e) published after 2000.

The review excluded studies from high income countries (HICs) [28]; focusing on paediatric cataract since the factors impacting patient decision-making are different; those in languages other than English, French and Portuguese; studies that reported cataract coverage rather than uptake of referral; qualitative studies, and those published before 2000.

### Study selection, data extraction and quality assessment

Two reviewers (EM and BV) independently screened the titles and abstracts, followed by full-text assessments against the inclusion criteria. Data extraction and quality assessment was conducted by the same reviewers independently. The Cochrane Public Health’s data extraction template was customized to reflect the research questions of this review [29]. Discrepancies were reconciled through discussions between the reviewers.

The methodological quality was assessed using an appropriate Critical Appraisal Skills Programme (CASP) checklist for each study design which ensured consistency [30]. Following the assessment, studies were attributed low risk of bias if they employed appropriate methods and reported study limitations. Medium risk was attributed if authors did not fully report the methodology or used a biased approach but acknowledged its limitations and did not draw strong policy conclusions. High risk was attributed if the authors used an ambiguously defined or biased approach and did not acknowledge its limitations.

## Results

### Search results

**Fig 1:**
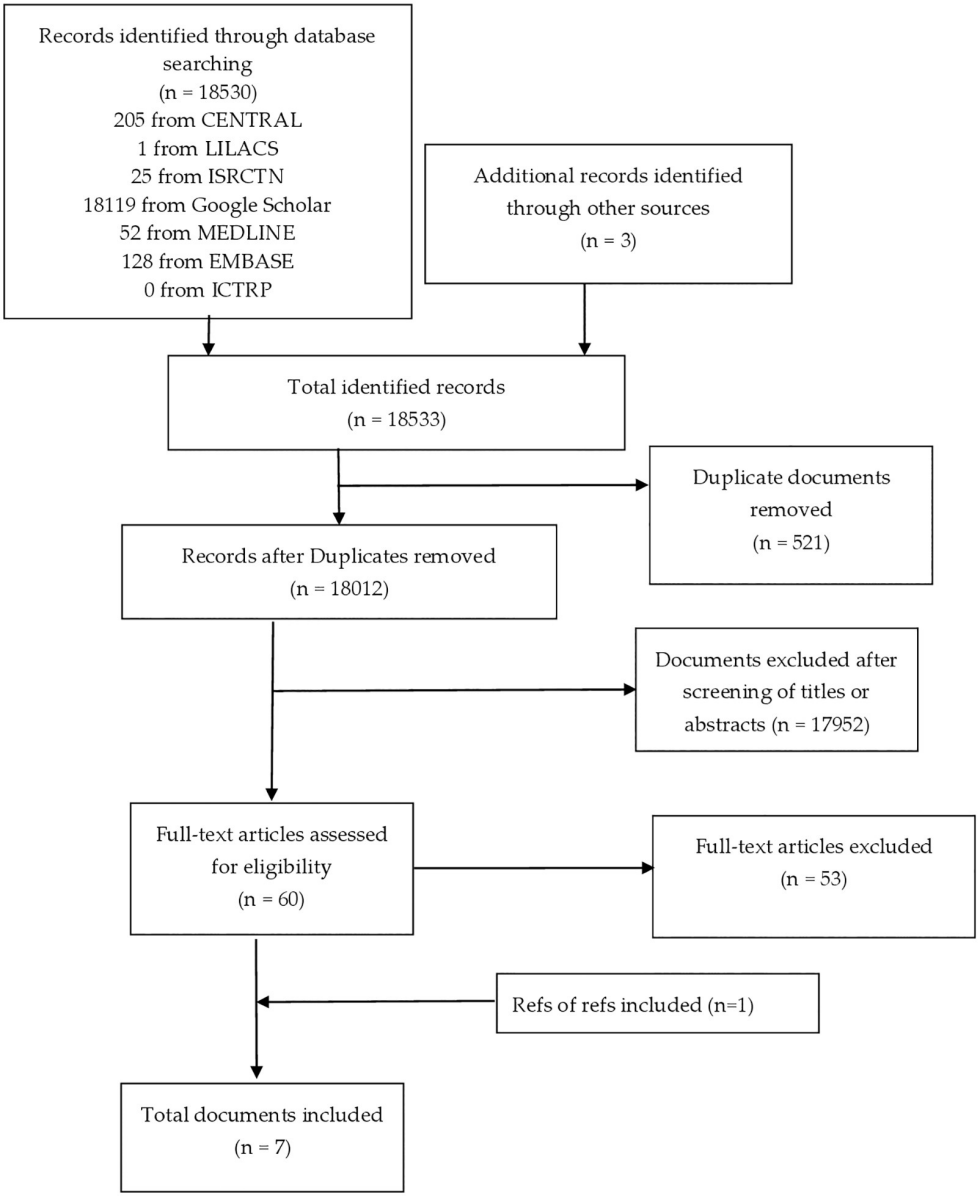
The Preferred Reporting Items for Systematic Reviews and Meta-Analyses (PRISMA) flow diagram for searching, screening and selection processes.

The database search yielded 18,530 records. After removing duplicates, 17,955 unique records were identified and subjected to a screening of titles and abstracts. Sixty studies were selected for full-text screening against the inclusion/exclusion criteria. Out of these, six were included in the review. One additional paper was identified and selected for inclusion through the references of the seven eligible studies [31]. As a result, a total of seven studies were included in the review (Fig 1). All studies were written in English and none was found in other languages.

### Description of included studies

Out of the seven included studies, two were intervention (RCTs) and five were observational, i.e. cross-sectional, and longitudinal surveys and prospective and retrospective cohort studies. Studies reported data from four Asian countries including China (n = 2); India (n = 2); Bangladesh (n = 1); and the Philippines (n = 1); and three African countries: Ghana (n = 1); Madagascar (n = 1); and Kenya (n = 1). Data from Kenya, Bangladesh and the Philippines were collected in one multi-country study [32]. All seven studies reported on both cataract surgery uptake and factors associated with the uptake but only two studies [33] reported interventions aiming to improve CSU (Table 1).

**Table 1:** Summary table of included studies.

Reference (author, year)	Location, participants, and sample size	Study design	Primary outcome; context; conditions of surgery	Surgery uptake	Study quality
**Liu et al., 2012**	China, Guandong. Patients 50+ years with cataract and PVA<6/18 recruited through screening in five rural hospitals; N = 212 intervention and N = 222 control.	Randomised Controlled Trial	Outcome: surgery in at least one eye 6 months or less after the screening Cost of surgery reimbursed by government insurance	31.1% intervention and 34.2% controls	Low risk of bias
**Xiu Juan Zhang et al., 2013**	China, Pucheng Shaanxi Province Patients 50+ years with operable cataract recruited through a screening programme across 24 towns; N = 541 identified and referred. N = 355 referred but did not take up referral, could be found and agreed to take part (73.8% (N = 262 women)	Prospective, randomized, intervention design.	Outcome: surgery within 3 months after the screening/ study recruitment A low-cost cataract surgery (240 RMB (~USD 38)) in the County Hospital (initial screening and group 1); free surgery in group 2; free surgery and reimbursement of transportation (after the surgery) in group 3; free surgery and free rides to and from the hospital in group 4. Reminders were sent in all groups 2 and 5 days after the referral.	20.1% initial (pre-intervention) (109/541) Post-intervention: 14.4% Group 1 (13 /86). 27.8% Group 2 (25/86) 31.1% Group 3 (28 /90). 28% Group 4 (26/93)	Low risk of bias
**Ackuaku-Dogbe, Yawson, & Biritwum, 2015**	Ghana. Rural and urban settings; Older adults 50+ years with self-reported diagnosed cataract recruited from the general population; N = 231 (124 female, 107 male).	A cross sectional survey of the nationally representative sample.	Outcome: self-reported surgery in the past five years. The context and conditions of surgeries is unknown.	48.9% overall (113/231); 48.4% women (60/124) 49.5% men (53/107).	Medium risk of bias
**Amritanand, Jasper, Paul, & Kuriakose, 2018**	Southern India Tamil Nadu Patients with operable cataract identified through an outreach programme (screening camps and clinics in primary and secondary facilities); N = 4682 and a subset of 196 bilaterally cataract blind patients who underwent surgery	A retrospective cohort study of cataract patients referred for surgery and a cross-sectional survey of bilaterally blind patients operated for cataract	Outcome: uptake of referrals over one year. Surgeries at a base hospital; patients were either referred or provided transport; surgeries performed at a subsidized rate or free of charge; patients also received free food at the hospital.	76.4% uptake of referral (3577/4682) 67.9% uptake of surgery (3178/4682)	Medium risk of bias
**Finger et al., 2011**	Southern India, Tamil Nadu Individuals aged 30+ years with cataract and corrected VA of <6 ⁄ 60 or those who were thought to benefit from surgery identified through outreach clinics following community mobilisation and door to door search of blind patients N = 1,045 in two districts (N = 797 and N = 248)	A retrospective study of a cohort of patients referred for cataract surgery	Outcome surgery 6 months or earlier after the referral. Five camps over 14 months in one district and eight camps over 6 months in another district. For those with a monthly household income < Rs. 1200 (approx. 25 US$;) surgery, inpatient stay, transport from and to hospital and eye drops post-surgery were provided free.	91.7% overall uptake 94.6% in district with regular outreach 82.3% in district with irregular outreach	Medium risk of bias
**Syed et al., 2013**.	Kenya, Nakuru district, Bangladesh, Satkhira district; the Philippines, Antique district, and Negros Island. Patients aged 50+ years with BCVA <6/24 in the better eye due to cataract identified through a population-based survey and case detection in the community N = 147 in Kenya, N = 217 in Bangladesh and N = 238 in the Philippines.	A longitudinal survey with one year follow up	Outcome: attendance for surgery 12 months after the referral. In Kenya and Bangladesh, surgery was free and transport expenses were reimbursed for all cases; In the Philippines, costs were subsidized for those who could not afford the fee. All cases were visited up to four times to encourage them to attend for surgery	58.6% in Kenya, 53.9% in Bangladesh 47.1% in the Philippines;	Medium risk of bias
**Razafinimpanana, Nkumbe, Courtright, & Lewallen, 2012**	Madagascar, Sava Region. Individuals aged 16+ years with cataract and VA <6/60 identified by case finders in the community	A prospective cohort study of patients with cataract	Outcome: attending hospital appointment next day after the screening. Patients recruited over 9 months period. Surgeries at SALFA Eye Clinic with the price for surgery adjusted according to a patient’s ability to pay	25.9% referral uptake (35/142) 24.6% surgery uptake (32/142)	High risk of bias

### Quality assessment of the included studies

Two studies, both RCT design [31, 33] were attributed low risk of bias. Four studies were attributed medium risk of bias [32, 34–36]. One cohort study [37] was attributed high risk of bias as authors did not justify the use of purposive sampling of villages, when they could have potentially used random methods, therefore introducing selection bias.

### Uptake of cataract surgery reported in LMICs

In two RCTs, the rates were reported for different study groups, two groups (intervention and control) in the Liu et al study [31] and four groups (three intervention and one control) in the Xiu et al study [33]. The multi-country study [32] reported CSU for each of the three studied countries.

The reported uptake rates varied greatly, from 14.4% in the control group in the RCT in North Western China [33] to 91.7% in a district outreach programme in Southern India [36]. The group of patients in China [33] included those who had already been referred to surgery earlier but did not take up the referral. If we use the initial screening data and the CSU from this study, it will still be the site with the lowest uptake rate with only 20.1% of patients taking up the surgery in the three months following the screening.

In both China RCTs [31, 33] and in the study in Madagascar [37] only a quarter to a third of patients offered a cataract surgery took it up. Two studies with the highest CSU (67%+) were both from southern India, where patients were identified through district outreach programmes. The remaining sites (Ghana, Kenya, Bangladesh and the Philippines) reported rates between 47% and 58% [32, 34].

The follow up period over which the referred patients were expected to attend surgery, also varied from 1 day to 12 months but it does not seem to have affected the CSU.

The reported CSU was disaggregated by sex in three studies [34, 36, 37].

### Factors that influence uptake of cataract surgery in LMICs

All seven studies reported factors associated with the CSU but statistical analyses to test the strength of the association was reported only in six studies. One study from Southern India [35] reported only descriptive statistics to describe factors influencing the uptake.

For the purpose of this review, factors reported in the studies (Table 2) were grouped under four categories: i) patients demographic and socio-economic characteristics [31–34, 36, 37] ii) costs of surgery and distance to the health facility [31, 32, 34, 36, 37]; iii) health status, quality of vision and awareness and history of cataract [32, 36]; and iv) perceived quality of services and opinion of others [35, 36].

**Table 2:** Analysis of factors associated with uptake.

Reference (author, year)	Factors tested for associations with %, RR OR CI p-values and all factors named in qualitative studies
**Liu et al., 2012**	**Significant associations with CSU (multivariate logistic regression model)**: Poorer Vision in the worse eye (worse logMAR PVA in the worse eye OR 1.61, 95% CI 1.22–2.12, P≤ 0.001 Knowledge of cataract surgery as the only way of treatment OR 1.83, 95% CI 1.07–3.13, P 0.05 Greater anticipated loss in income from going to hospital OR 1.36, 95% CI 1.01–1.83, P 0.05 Greater floor space per person in house OR 1.27, 95% CI 1.09–1.47, P 0.01 **No association**: Younger age 1.02 (0.99–1.04); Female sex 1.26 (0.76–2.08); Family member accompanied to screening 1.44 (0.87–2.39); Believes vision will improve ‘‘a lot” after surgery 1.40 (0.82–2.40); Thinks surgeons at hospital are ‘‘highly skilled” 1.40 (0.81–2.42); Thinks doctors and nurses at the hospital have ‘‘very good” attitudes 1.99 (0.97–4.08); Greater anticipated spending on food/lodging 1.01 (0.74–1.37); Exposure to health education intervention 1.11 (0.67–1.84) No univariate association not included in multivariate logistic model): Received some formal education 1.29 (0.85–1.97); Presenting logMAR vision in better eye (worse) 1.06 (0.87–1.31); Screening during summer 0.96 (0.35–2.64); Previous cataract surgery 1.18 (0.70–2.00); Knows someone who had cataract surgery 0.90 (0.59–1.37); Thinks cataract can be treated 0.98 (0.56–1.73); Believes surgery will hurt 1.24 (0.81–1.92); Greater anticipated out-of-pocket payment for surgery 0.93 (0.59–1.45); Self-pay 0.83 (0.48–1.45); Greater anticipated spending on transportation 1.20 (0.98–1.47); Less than 1 hour’s travel from hospital 1.40 (0.78–2.49); Family member available to accompany to hospital 1.28 (0.71–2.32); > 100 days between screening and scheduled hospital examination 0.64 (0.29–1.44)
**Xiu Juan Zhang et al., 2013**	**Significant association with CSU (Chi-square test for bivariate analysis)**: Male sex (men 32/93, 34.4%; women 60/262, 22.9% P 0.03) **No association**: Age, 50–59: 35%, 60–69:24.8%; 70–79: 26.1%, 80+ 23% P 0.788 Education: none 22.6%; 1+ years 29.2% P 0.155 Vision: <6/50 25.2%; <6/18 >6/60 27.4% P 0.656
**Ackuaku-Dogbe, Yawson, & Biritwum, 2015**	**Significant association with CSU (Chi-square test for bivariate analysis)**: Availability of health insurance (54.7% vs 42.1%, P 0.055) **No association**: Sex, women 48.4%, men 49.5% p 0.867 Age, 50–59: 34.2%, 60–69: 55%; 70+: 49.6%, P 0.123 Residence: Urban 52.6%; Rural 44.3% P 0.21 Marital status: with partner 47.7%; without partner 50% P 0.6 Education: Primary or less 56.3%; Secondary and above 57.6% P 0.886 Income quintile Q1, Q2 and Q3 (lower) 47.7%; Q4,5 (higher) 50% P 0.364 **Not tested statistically**: Regions: Ashanti 45.5%; Brong Ahafo 66.7%; Central 44.8%; Eastern 56.7%; Greater Accra 51%; Northern 40%; Upper East 20%; Upper West 14.3%; Volta 58.3%; Western 40.7%. P not reported
**Amritanand, Jasper, Paul, & Kuriakose, 2018**	**Frequency of reported facilitating factors (no statistical analysis reported)**: Neighbours and acquaintances (48/168, 28.6%); health care staff (34/168, 20.2%); persons who have undergone cataract surgery (33/168, 19.6%); family members (27/168, 16.1%); mass announcements (19/168 (11.3%); location of the camp near home (7/168(4.2%).
**Finger et al., 2011**	**Significant associations with CSU (binary logistic regression model)**: Study area (regular vs irregular screening), p = 0.003 Had already had surgery in one eye, p = 0.003 **No associations**: Age, p = 0.371 Sex, p = 0.531 Visual acuity in the better eye, p = 0.092 **No univariate association, and not included in binary logistic regression model**: Self-reported co-morbidity Schooling Household size Monthly income Occupation
**Syed et al., 2013**.	**Significant associations with CSU: (Multivariate model)**: **Kenya** Sex: Male v Female (baseline) AOR 2.6 (95%CI 1.1–6.4) Age: 50–69 v 70+ (baseline) AOR 5.0 (95%CI 1.3–20.2) Psychosocial score: Lowest v highest (baseline) AOR 2.2 95%CI 1.2–8.8); Middle v highest AOR 2.9 (95%CI 1.1–8.3); VA in better eye <3/30 v ≥ 3/60 (baseline) AOR 4.4 (95%CI1.8–10.6); **Bangladesh**: Sex: male v female (baseline) AOR 3.0 (95%CI 1.6–5.5) Age: 50–69 v 70+ (baseline) AOR 2.8 (95%CI 1.5–5.4) Psychosocial score: lowest v highest (baseline) AOR 2.2 (95%CI 1.1–4.7) **The Philippines**: Age: 50–69 v 70+ (baseline) AOR 2.1 (95%CI 1.2–3.7) **No associations**: **Kenya**: Marital status: married v not married (baseline) AOR 1.3 (95%CI 0.5–3.1) **Bangladesh**: Location: Rural v urban (baseline) AOR 6.0 (95%CI 0.7–55.2) Psychosocial score: Middle v highest (baseline) AOR 1.1 (95%CI 0.6–2.2) **The Philippines**: none reported **No univariate associations and not included in multivariate model**: **Kenya**: location; marital status; case type; work in the last week; literacy; SES score; overall eyesight rating; general functioning score; self-rated health **Bangladesh**: VA in better eye; marital status; Case type; work in the last week; literacy, SES score; overall eyesight rating; general functioning score; self-rated health **The Philippines**: Sex; marital status; VA in better eye; location; work in the last week; literacy; SES score; overall eyesight rating; general functioning score; psychosocial score; self-rated health
**Razafinimpanana, Nkumbe, Courtright, & Lewallen, 2012**	**Significant association with CSU (Multivariate model)**: Being from a nearby district of Sambava: Relative Risk, RR (95% CI) = 1.8 (1.1–3.2); p = 0.04. Money for transport, p = 0.006 (no RR, reported as bivariate) Money for both transport and food, p = 0.004 (no RR, reported as bivariate) **No association**: Female sex, RR (95% CI) 1.1 (0.6–1.9); p = 0.9 Being married, RR (95% CI) 0.9 (0.5–1.7); p = 0.9 Being literate, RR (95% CI) 0.9 (0.5–1.6); p = 0.6 Had visited Sambava before, RR (95% CI) 0.8 (0.4–1.7); p = 0.5 Has a relative in Sambava, RR (95% CI) 1.0 (0.5–1.7); p = 0.9 Know price of surgery, RR (95% CI) 1.4 (0.5–3.7); p = 0.4 Had previous surgery, RR (95% CI) 1.1 (0.4–3.1); p = 1.0 Know another person having cataract surgery, RR (95% CI) 1.5 (0.8–2.6); p = 0.3 Age, p = 0.71 Number of children alive, p = 0.38 Money for food at hospital, p = 0.3 Money patient is willing to pay for cataract surgery, p = 0.9

#### Demographic and socio-economic characteristics

*Gender*. Sex was explored as an influencing factor in six studies [31–34, 36, 37). Out of these, three sites (China, Kenya and Bangladesh) [32, 33] found significant differences in CSU between men and women and in all three, the uptake of surgery was higher among men.

In four settings, China [31], the Philippines [32], Southern India [36] and Ghana [34] there were no statistically significant differences in the CSU by gender.

*Age*. Six studies explored age as a predictor of CSU [31–34, 36, 37]. Statistically significant associations between age and the CSU were found only in the multi-country study [32] in Bangladesh, Kenya, and Philippines with all three sites reporting that relatively younger patients (50–60 years) were more likely to take up surgery than those of older age (>70 years) (OR 2.8, 1.5–5.3; OR 6.2, 1.7–22.8 and OR 2.1, 1.2–3.7 respectively). Studies in China [31, 33], Ghana [34], Southern India [36] and Madagascar [37] found no association between age (regardless of age categorisation) and CSU.

*Marital status and family*. Six studies examined the role of marital status and family more broadly [31, 32, 34–37]. Three studies [32, 34, 37] examined whether being married was associated with CSU and found no statistical associations. The study in Madagascar, also examined whether the number of children alive was associated with CSU and found no significant associations. Finger et al found no relationship between the household size and the CSU; while Liu et al explored whether having someone to accompany screening or to hospital was associated with the CSU and found no statistical association. In the study in Southern India [35], the opinion of family members was rated to be the fourth out of six factors influencing the CSU reported by 16.1% of participants.

*Education and occupation*. Five studies [32–34, 36, 37] explored the role of education and patient literacy in the surgery uptake. None of the studies showed statistically significant associations. Occupation or work in the past month was examined in two studies [32, 36] with no statistically significant associations found.

*Income and wealth*. To assess the role of wealth, studies used various variables, including monthly income [36], socio-economic score [32] or quintile [34] and floor area per household member [31]. Only one study from China showed that greater floor space per person in the house was associated with higher surgery uptake (OR 1.27, 95% CI 1.09–1.47, P< 0.01). There were no statistically significant associations found in other studies.

*Residence*. Four sites in Ghana, Kenya, Bangladesh and the Philippines explored whether living in urban versus rural areas was associated with CSU; no statistically significant differences were found in any of these settings [34]. The study from Ghana also explored regional differences in CSU; it reported that the uptake of surgery was considerably lower in the Upper East and Upper West regions (20% or less) compared to other regions (40% or more). However, no results of the statistical analysis by region were reported in the paper.

*Cost of surgery and distance to health facilities*. In this section we explore the role of direct and indirect cost of surgery, including user fees, associated costs of travel, food, and lost opportunity income. We also examine whether the availability of health insurance was associated with higher CSU. A total of five studies explored the role of these factors.

Overall, information on cost of surgeries and associated expenditures was limited in the studies reviewed. Only two sites (Kenya and Bangladesh) reported that the surgeries provided were free for all patients [32]. In the majority of sites, surgeries were provided at a discounted rate but there was no information on either the sliding scale used or the proportion of patients entitled to the discount. Similarly, trasnport to and from the hospital, food at the hospital and post-surgery medicines were provided for free to some patients in some sites but there was little information on who benefited from these entitlements and who did not. Liu et al in China argued that removing user fees inflates the uptake of surgery and required that fewer than 50% of patients included in the trial received surgeries for free. The same study however reported that patients were entitled to the government insurance scheme but there was no further information of who was covered under this scheme and for which services [31]. The study in Ghana reported no information on the terms and conditions of the cataract services provided [34].

Only one study (an RCT by Xiu et al) specifically investigated the impact of user fees removal on the CSU. The study found that provision of free cataract surgery significantly increased the CSU (27.8% vs 14.4%, P = 0.027). Uptake of surgery in the groups with free surgery and reimbursement or provision of transport was also statistically significantly higher than in the reminder and low cost surgery group (31.1% vs 14.4% (P = 0.012) and 28% vs 14.4% (P = 0.038)). However, when compared to the free surgery only, reimbursement of transport fees or free rides to the hospital did not make a significant difference for the CSU (*P* = 0.768; and *P* = 0.869, respectively) [33].

Three other (observational) studies investigated the impact of direct and indirect costs on CSU. Liu et al in China found no association between CSU and anticipated costs of surgery, self-pay, anticipated spending on transportation or distance to hospital. The only variable that showed association with CSU was anticipated loss of income due to hospitalisation, but this association was relatively weak (OR 1.36, 95% CI 1.01–1.83, P = 0.05). In the descriptive study in Southern India [35], only 4.2% of cataract patients said that the location of the outreach camp near their home impacted on their decision about the surgery.

In contrast, in Madagascar [37], people living closer to a health facility (Sambava district) were more likely to present for surgery than those from more distant districts (RR (95% CI) = 1.8 (1.1–3.2); P = 0.04). However, having a relative in the district, where the hospital was located or having visited the district before was not associated with CSU. Also, there was no statistically significant associations between CSU and anticipated costs of food or patient willingness to pay for surgery itself. However, the lower anticipated costs of transport and transport and food combined were associated with the higher CSU.

The study in Ghana compared CSU among people with and without medical insurance. The uptake of surgery among people covered by insurance was higher than for those who were not covered (54.7% and 42.1% respectively, P = 0.055) [34].

*Health status*, *quality of vision and awareness and history of cataract*. Several studies examined the relationship between CSU and patient general health, quality of vision and knowledge and experience of cataract. The association between CSU and poorer vision was explored in three studies. Two studies, in China and India, [33, 36] found no statistically significant associations. Another study in China [31] found that CSU was higher among patients with poorer vision in the worse eye but not in the better eye. Syed et al [32] found that CSU was higher among patients with poorer vision in the better eye but only in Kenya. The same multi-country study explored the relationship between CSU and other self-reported aspects of vision, i.e. eyesight rating and functioning score but found no difference in any of the three sites.

Knowledge of cataract was examined in one study in China [31], which found that only knowledge of surgery as the only treatment of cataract was associated with the higher CSU. The same study examined whether knowing someone operated for cataract increased CSU and found no significant associations. In the study in Southern India [35], 19.6% of cataract patients said that the opinion of other patients, who had undergone cataract surgery influenced their decision about the surgery but only one in ten (11.3%) regarded mass media announcements, as an important source of information for their decision about cataract.

Previous experience of cataract surgery was explored in three studies [31, 36, 37]. Only the study in Southern India found that having been operated for cataract increased CSU (P = 0.003). There were statistically significant associations in the other studies.

Other aspects of patient general health were measured in two studies, including self-reported co-morbidities [36], self-rated health and psychosocial scores [32]. There were no associations between self-reported health or co-morbidities and CSU [32, 36]. The association between psychosocial scores and CSU were reported in Kenya and Bangladesh but the directions of the association were reverse. In Kenya, CSU was higher among patients with better psychosocial scores; in Bangladesh, the uptake was higher in patients with worse psychosocial scores [32].

*Perception of cataract services*. A number of studies explored whether perception of cataract services, opinions of others or how cataract services were delivered influence CSU. Two studies from China found that neither patients’ perceptions of health providers’ attitudes and skills, nor their fears that surgery may hurt, nor the delivery of surgery in summer season influenced the uptake [31, 33]. Only the study from Southern India found that the delivery of services in the districts with regular outreach programmes increased the uptake rates compared to the districts with more ad hoc cataract provision [36]. The descriptive study from Southern India [35] found that over 28% of cataract patients were influenced by the views of their neighbours and acquaintances about the surgery, while one in five (20.2%) relied on the opinion of health care staff.

### Interventions that have been reported to improve uptake of cataract surgery in LMICs

Only two studies, both from China tested specific interventions to improve CSU (Table 3). The quality of evidence from these two studies was rated high. Lui et al [31] randomly allocated 434 patients from 26 villages or townships to the intervention and control groups. The intervention group received an informational video and a group education/counselling. The study found that the exposure to education about cataract surgery did not increase the surgery uptake (OR = 1.11, 95% CI 0.67–1.84).

**Table 3:** Summary of included randomised trials.

Reference (author, year)	Interventions tested	Interventions effect
**Liu et al., 2012**	5-minute informational video about cataract and cataract surgery, which consisted first of segments of an interview in the local dialect with a cataract patient and family members describing the impact of the patient’s reduced vision on family life before surgery.	Intervention had no effect, no statistically significant difference on uptake of cataract surgery. 31.1% intervention and 34.2% controls (P>.0.5)
**Xiu Juan Zhang et al., 2013**	Group 1 Informative reminders by telephone or in person by a trained facilitator about undergoing low-cost cataract surgery. Group 2 In addition to the reminders, offered free cataract surgery. Group 3 In addition to the reminders and free surgery, offered reimbursement of transportation expenses (after the surgery). Group 4 In addition to the reminders and free surgery, offered free rides to hospital and back.	Introducing free surgery significantly increased the uptake of surgery compared to the reminders and low-cost surgery. Uptake of surgery in the groups with free surgery and reimbursement or provision of transport was also statistically significantly higher than in the reminder and low-cost surgery group. Group 1 14.4% Group 2 27.8% (*P* = 0.027) Group 3 31.1% (P = 0.012) Group 4 28% (P = 0.038) However, introduction of transport cost reimbursement or free rides to hospital did not make a significant difference for the uptake compared to the free surgery only. Groups 2 and 3 (*P* = 0.768) Groups 2 and 4 (*P* = 0.869)

The other RCT consisted of three intervention and one control groups. Patients enrolled in the trial included those, who had been earlier referred but did not attend the surgery in the three months since the referral. Group 1 (86 participants, control) received surgery reminders only; Group 2–86 participants in addition to the reminders, were offered free surgery; Group 3–90 participants were also offered reimbursement for transport; and Group 4–93 participants were offered free rides to and from the hospital. CSU in group 2 was significantly higher than that in group 1 (P = 0.027). However, there was no significant differences in CSU between groups 2, 3, and 4. The study concludes that the provision of free surgeries, but not necessarily additional free transport increases the surgery uptake.

## Discussion

This systematic review aimed to synthesise all available evidence on the uptake of cataract surgeries in LMICs, including reported uptake rates, factors influencing the uptake and interventions reported to improve it. The review builds on two other systematic reviews [23] and [24], which investigated interventions to improve equity in eye care. Although this study expands upon the findings of those reviews by including studies with a broader range of methodological approaches, the lack of robust intervention studies remains a major weakness in this area.

Many studies that measured access to cataract services using standardised approaches like RAAB or its predecessor, RACSS (Rapid Assessment of Cataract Surgical Services), highlighted low coverage in many LMICs. In many settings, however, this low coverage is often assumed to be a function of the limited infrastructure and human resources. In this review, we focused on surgery uptake, which mitigated the impact of these factors. In all studies included in the review, patients were offered a surgery, which means the system had sufficient capacity to meet their demand, and the decision was largely down to the patient, although, as evident from our review, there is a considerable degree of overlap between provider (supply) and patient (demand) level factors.

The review includes both intervention and observational studies and suggests that the uptake of cataract surgery varies greatly between the settings and depends on population characteristics, cataract programme set up and the context, in which it is being delivered. Evidence shows that stimulation of demand for cataract surgeries is critical for maximising the effectiveness and efficiency of cataract services [12, 38]. Programmes that do not manage to achieve the optimum uptake rates often waste a significant part of their resources on patient mobilisation and screening, which do not result in sight restoring surgeries and reduction of avoidable visual impairment [39, 40].

Our findings show that demand for cataract surgery is influenced by factors at individual, community, and health system levels. At the individual level, the most consistent finding across the settings is gender-related inequalities. The disadvantages in women’s access to eye care services have been well documented and are often attributed to the inferior social and economic position of women found in many settings [36, 41–45]. This socio-cultural perspective presents men as superior to women to an extent that men’s health is prioritized more than women’s health by both men and women [16]. In addition, women who are traditionally the primary family carers don’t have time to access services due to their demanding household duties; and in many settings, they are neither allowed nor have economic means to travel and seek care away from their communities. Being house-bound makes women’s access to health information and care limited [43, 46, 47]. One aspect that needs to be considered is that in a number of studies in this review, cataract surgery uptake was measured for those who presented at screening camps. In many settings, many women do not even get to the camps; therefore, this review may underestimate gender inequities present in cataract care.

Findings on age as a factor impacting the CSU suggest that relatively younger population sub-groups (50–60 years) show higher cataract uptake. This may be related to the higher value of sight in younger populations, who may be less likely to have other morbidities or benefit more from being economically productive and mobile. It may also be determined by specific programme contexts, for example, how easy it is to get to the hospital or what support structures older people have in their families. Either way, programmes need to be aware that older age may impact the uptake of cataract surgery irrespective of other factors influencing the demand.

An interesting finding was the lack of association between CSU and marital status or broader family support. This is in contrast with cross-sectional surveys, which measure cataract coverage at the population level and often show that single people, particularly women living alone and having no companions to go to screening and hospitals with, tend to be the ones left out of the cataract services [38, 48]. Qualitative studies [12, 49] also suggest that the availability of family support and someone to escort are the key factors motivating patients to take up surgery. Family support can be complex and difficult with increasing household size and is experienced differently by men and women [36, 41, 50, 51]. The role of the family in CSU requires further investigation and more specifically, how the family dynamics plays out in different cultures and contexts. A lack of reliable and valid survey instruments designed to effectively measure family support across different cultures or contexts may also explain the lack of evidence observed in quantitative surveys.

Further, other studies show that the opinions of others in the community, neighbours, acquaintances and friends, is an important facilitator to CSU [50, 52]. The descriptive study included in our review showed the same [34]. Studies that applied regression models did not show an independent significant effect of knowing someone operated for cataract on CSU. The explanation for this could be methodological, as most studies had relatively small sample sizes and the tools used to ascertain these data were not standardised or validated for local contexts. To better understand the role of different community level factors, such as family support, local cultural norms and perceptions of local surgical services, we need larger quantitative studies and analyses of large programme data sets using more refined and culturally tested data collection tools.

There are mixed findings on access to cataract surgery among people in lower socioeconomic groups. Available literature is also often inconsistent [10, 53]. However, these results are likely to be undermined by a range of methodological and contextual factors. First, socioeconomic status is a complex issue determined by several very culturally specific issues that are hard to boil down to a simple tool. Second, many published studies of cataract services take place in poor rural locations with patients recruited through outreach services, which often target the poorest segments of the population. In these contexts, all patients tend to be poor and even the tools designed to measure relative wealth often fail to detect the difference. In addition many eye care programmes supported by iNGOs provide free or subsidised services, which proactively target the poor and it is difficult to establish the true relationship between the socio-economic status of patients and their demand for services in such contexts.

Our findings broadly support the argument that the removal of patient direct and indirect costs can improve the surgery uptake, although the influence of these factors is likely to be nuanced depending on the local context. Contextual factors, such as population density and local transport infrastructure are likely to be important mediators of the relationship between the costs of surgery and surgery uptake. In addition, some studies in this review show that even when free surgery is provided, the uptake may not be as high as one would expect [32]. It remains unclear whether demand for cataract surgery in such contexts is influenced by indirect costs of food, transport and time or more intangible costs of anxiety and fears associated with hospitals and surgery itself. [17, 54–57]. These findings call for a more in depth analysis of the surgery uptake data in the context of services available locally, using both qualitative methodologies to supplement quantitative data [10].

Regular outreach was reported to improve uptake of cataract surgery. This is consistent with qualitative studies conducted in India [36], Tanzania and Kenya [40]. This could mean that outreach was used as a mechanism to overcome the difficulties of distance, and its associated transport cost [42, 58, 59]. This may also mean that there is a particular pattern of service delivery patients get used to and this familiar way of receiving services stimulates the demand. Such strategies are often used in marketing to influence consumer behaviour [60, 61]. Evidence on the role of such factors in healthcare is less clear [10] and further research in this area is needed.

In contrast to other studies of demand for health services [62], this review did not provide evidence of the association of patient education and CSU. It may be due to the fact that cataract affects primarily older people and in the contexts where these studies were conducted most patients in this age group have low level of education and similarly to the socio-economic status, it would be difficult to capture the difference. Our review however suggests that raising knowledge of surgery as a treatment for cataract does increase the uptake. Adequate counselling of patients as a way to address patient fears and reduce the likelihood of refusals has been highlighted in other studies [58, 63]. The use of successfully operated patients as champions and councillors has also been identified as an important strategy [35, 41, 50, 58]. Although our review, does not provide evidence on the relationship between knowing someone operated for cataract and surgery uptake, this area of research needs to be investigated further. This strategy is contingent upon the operated patients reporting positive experiences of both the outcome of the surgery, and the process at the health facility. Perceptions of quality of clinical and non-clinical care have been identified in other studies as associated with uptake of services and it is important to note that patient perceived positive outcomes do not always align with objectively measured clinical standards [59, 64, 65].

An important finding of this review was the glaring lack of evidence on the strategies that work in LMICs to improve CSU. The finding calls for an urgent need to conduct intervention studies that test different approaches to improve surgery uptake and their effectiveness. It is also critical that these studies are of sufficient size to be able to examine associations between outcomes and patient characteristics to understand who benefits from these approaches and who does not. Studies focusing on the uptake of surgery by specific population subgroups are also needed. Poor uptake of cataract surgeries by women needs to be addressed as a matter of priority, as evidence on women’s disadvantage in accessing cataract services is rather consistent [46].

A number of limitations of this review need to be considered. First, although authors conducted a thorough search of the literature, this review cannot preclude the presence of publication bias, therefore sources included in this review may not be representative of all available information on this subject. This review may also be prone to language bias as it only searched for studies written in English, Portuguese and French, although no studies written in other languages were identified.

Overall, included studies were mainly observational studies with medium and high risk of bias, as methods used were not clear or were not reported. Evidence on the impact of strategies to improve cataract surgery uptake was limited to two studies, both from China. Further research should focus on using robust methodologies to measure the effect of particular interventions to improve uptake, particularly among disadvantaged groups. Opportunities for conducting this sort of study may need to be developed in partnership between academic researchers and iNGOs who are often responsible for providing financing of eye health programmes in LMIC [66].

Further, a large number of studies included in the review reported findings from outreach services. The limitation of this is that most patients identified through outreach are rather homogenous with respect to their characteristics, which may explain why statistically significant differences were not identified for important factors such as education, socio-economic status, or residency.

Finally, the studies identified in this review focused primarily on demand-side interventions. Further studies exploring the effect of supply-side factors will be needed.

In conclusion, the review shows that the uptake of cataract surgery by patients varies greatly between settings and there is a variety of factors impacting patients’ demand. It also highlights a lack of quality intervention studies. Improving the uptake of cataract surgery in LMICs will require development and testing of strategies that stimulate the demand and maximise the efficiency and equity of current and future cataract programmes, particularly among disadvantaged groups, such as women.

## Supporting information

S1 Appendix (DOCX)
