# Supplementary material for: Factors associated with the uptake of cataract surgery and interventions to improve uptake in low- and middle-income countries: A systematic review
Source: PLoS One. 2020 Jul 9;15(7):e0235699. doi: 10.1371/journal.pone.0235699 (PMC7347115; doi:10.1371/journal.pone.0235699)
Supplement: S1 Appendix — (DOCX) [file pone.0235699.s001.docx]

**Databases and search words**

| **Databases/websites** | **Search words** |
| --- | --- |
| Embase Classic (Ovid) | 1. Cataract 2. Surgery OR treatment 3. Uptake OR increase 4. Intervention OR facilitators OR effects OR service OR outcomes OR outputs OR management OR program OR project 5. Developing country 6. Africa 7. Asia 8. Low-income country 9. Medium-income country 10. China 11. 1 AND 2 AND 3 12. 5 AND 6 AND 7 AND 8 AND 9 AND 10 13. 11 AND 12 |
| Embase: Excerpta Medica (Ovid) |  |
| CENTRAL (***Cochrane Controlled Register of Trials)*** | cataract surgery AND developing countries |
| LILACS | cataract surgery uptake AND developing countries AND facilitators OR interventions |
| ISRCTN | cataract surgery uptake AND developing countries AND facilitators OR interventions |
| ICTRP | cataract surgery uptake AND developing countries AND facilitators OR interventions |
| University of Birmingham all search | cataract surgery uptake AND developing countries AND facilitators OR interventions |
| Google scholar | cataract surgery uptake AND developing countries AND facilitators OR interventions |
